# An updated checklist to the biodiversity data of ladybeetles (Coleoptera: Coccinellidae) of the Azores Archipelago (Portugal)

**DOI:** 10.3897/BDJ.9.e77464

**Published:** 2021-12-16

**Authors:** António Onofre Soares, Isabel Borges, Hugo Renato Calado, Paulo A. V. Borges

**Affiliations:** 1 IUCN SSC, Ladybird Specialist Group, Ponta Delgada, Azores, Portugal IUCN SSC, Ladybird Specialist Group Ponta Delgada, Azores Portugal; 2 cE3c – Centre for Ecology, Evolution and Environmental Changes / Azorean Biodiversity Group and Universidade dos Açores, Rua Madre de Deus, sn, Ponta Delgada, Azores, Portugal cE3c – Centre for Ecology, Evolution and Environmental Changes / Azorean Biodiversity Group and Universidade dos Açores, Rua Madre de Deus, sn Ponta Delgada, Azores Portugal; 3 cE3c – Centre for Ecology, Evolution and Environmental Changes / Azorean Biodiversity Group and Universidade dos Açores, Rua Capitão João d’Ávila, São Pedro, 9700-042, Angra do Heroísmo, Azores, Portugal cE3c – Centre for Ecology, Evolution and Environmental Changes / Azorean Biodiversity Group and Universidade dos Açores, Rua Capitão João d’Ávila, São Pedro, 9700-042 Angra do Heroísmo, Azores Portugal; 4 IUCN SSC, Mid-Atlantic Islands Specialist Group, Angra do Heroísmo, Azores, Portugal IUCN SSC, Mid-Atlantic Islands Specialist Group Angra do Heroísmo, Azores Portugal

**Keywords:** Arthropoda, Ladybeetles, Azores, Faial, Graciosa, Pico, São Jorge, São Miguel

## Abstract

**Background:**

A recently-published review from 2021 presents a comprehensive checklist of ladybeetles of Portugal, including the Azores and Madeira Archipelagos. Until then, the available information was very scattered and based on a single revision dating back to 1986, a few international catalogues and databases, individual records and studies on communities of agroecosystems. However, no information was available on faunal composition across the Azorean islands and their habitats, using standardised inventories. Here, we present data about the biodiversity of ladybeetles and their distribution and abundance in five Islands of the Azores (Faial, Graciosa, Pico, São Jorge and São Miguel). Surveys included herbaceous and arboreal habitats from native to anthropogenic-managed habitats: ruderal road vegetation, vegetable garden, mixed forest of endemic and non-native host plants, coastal prairies, coastal mixed vegetation, cornfields and urban areas. We aimed to contribute to the ongoing effort to document the terrestrial biodiversity of Portugal, including the Archipelago of the Azores, within the research project AZORESBIOPORTAL–PORBIOTA (ACORES-01-0145-FEDER-000072).

**New information:**

In this study, a total of 1,487 specimens of Coccinellidae belonging to 19 species are reported for several habitats. The listed species are from one single sub-familiy (Coccinellinae) and six tribes; Chilocorini (one species), Coccidulini (three species), Coccinellini (six species), Noviini (one species), Scymnini (seven species), Stethorini (one species). The number of species collected per island differed; Faial (10 species), Graciosa (four species), Pico (seven species), São Jorge (seven species) and São Miguel (12 species). For six species, new island records are given. Currently, the number of species known to occur in the Azores are 32, including two doubtful records. The majority of species are Scymnini, being Scymnus (Scymnus) interruptus (Goeze, 1777) and Scymnus (Scymnus) nubilus Mulsant, 1850, the most abundant species (relative abundance 71.1%). This database will be the baseline of a long-term monitoring project allowing assessment of the impact of ongoing global changes in the distribution and abundance of ladybeetles.

## Introduction

Insects, like other taxonomic groups, are at high risk of extinction (Harvey et al. 2020). Insects deliver fundamental services to agricultural and forest ecosystems, including pollination, decomposition and pest control, which, in turn, translates into relevant consequences for food production and security (e.g. Ameixa et al. 2018, IPBES 2019, Cardoso et al. 2020).

The family Coccinellidae contains between 6000 and 7000 described species (Seago et al. 2011). Currently the number of Coccinellidae known for Azores is 32, including two doubtful records (Soares et al. 2021b).

Despite being very diverse in terms of morphology, life history traits, habitat use and food relationships (see Hodek et al. 2012 for review), they are primarily top carnivorous predators and thus useful natural enemies of herbivorous arthropods, including aphids (Aphidoidea), scale insects (Coccoidea), whiteflies (Aleyrodoidea) or mites (Acari) (Hodek et al. 2012). Until very recently, this group was thought to exhibit only sexual reproduction. However, it was found that some populations of Nephus (Nephus) voeltzkowi Weise, 1910, including the Azorean populations, showed parthenogenetic reproduction, which constitutes the first case of parthenogenesis in ladybeetles (Magro et al. 2019).

Over the past 30 years, rapid declines of formerly common native ladybird species - including in North America (Harmon et al. 2006), Europe (Roy et al. 2012, Honĕk et al. 2016) and others (reviewed in Roy et al. 2016) - have been occurring. Most declines are associated with climate change, agricultural intensification and urbanisation and invasions of alien species (Honĕk et al. 2017), especially with an increasing density, spread and dominance of the invasive *Harmoniaaxyridis* Pallas. Despite its high invasive capacity resulting in its rapid spread and fast establishment under distinct climatic conditions, *H.axyridis* did not establish in the Azores where it was intentionally and repeatedly released (Soares et al. 2008, Soares et al. 2018), for the same reasons as in other regions, for agricultural pest control purposes. This apparent failure is an interesting case study for invasion biology. Several hypotheses were tested to explain the inability of this species to become invasive (Soares et al. 2017, Alaniz et al. 2020). The lack of high density of their preferred aphid preys may be a key factor hampering its establishment. Indeed, the composition of Coccinellidae fauna seems to be dominated by small species [like *Scymuns* spp., which require low aphid density (Soares et al. 2017)]. Apparently, the climatic conditions of the Azores do not seem likely to hinder the invasion of *H.axyridis*, as areas with similar climates have experienced extensive invasion. Indeed, climatic models have predicted the spread of *H.axyridis* to regions with subtropical conditions (Poutsma et al. 2008, Bidinger et al. 2010). However, for the Azores and contrary to that prediction, the absence of suitable temperature to overwinter will force adults to become active during the winter season and females will not find enough suitable food (in quantity and quality) to reproduce and this will hinder the build-up of the first generation (Alaniz et al. 2020).

## General description

### Purpose

We aimed to contribute to characterise the richness and abundance of ladybeetles in several herbaceous and arboreal habitats, from native to anthropogenic-managed habitats. We also aimed to contribute to address two key shortfalls: i) the need for improving current information on the local and regional distribution of Azorean arthropods (the Wallacean shortfall); and ii) the need for collecting abundance data for future monitoring purposes (the Prestonian shortfall) (see Cardoso et al. 2011).

In addition, we provide an updated checklist of Azorean ladybeetles with their known distribution in the nine Azorean islands.

## Project description

### Title

AZORESBIOPORTAL–PORBIOTA: inventory of ladybeetles of the Azores (Portugal)

### Personnel

António O. Soares, Isabel Borges and Hugo R. Calado collected the samples and managed the database. Paulo A.V. Borges assisted us in managing the database to GBIF.

### Study area description

We focused the inventory on five islands of the Azores (Table 1), these being five of the nine islands from the Azores Archipelago. The climate in the Azores is temperate oceanic, with regular and abundant rainfall, high levels of relative humidity and persistent winds, mainly during winter and autumn seasons. The landscape of the islands is composed by a mosaic of habitats, ranging from herbaceous to arboreal habitats and from native to anthropogenic-managed habitats. The surveys were done on ruderal road vegetation, vegetable garden, mixed forest of endemic and non-native host plants, coastal prairies, coastal mixed vegetation, cornfields and urban areas.

### Funding

This study was financed by FEDER in 85% and by Azorean Public funds by 15% through the Operational Programme Azores 2020, under the following projects AZORESBIOPORTAL–PORBIOTA (ACORES-01-0145-FEDER-000072) and under the project ECO^2^-TUTA (ACORES-01-0145-FEDER-000081) and by the Official Forestry Services from the Regional Government of the Azores, through the research projects PICA (Utilização de agentes de controlo biológico para o combate a populações de afídeos em plantas endémicas produzidas em viveiro) and PICONIA (Controlo biológico de populações de pragas de plantas endémicas produzidas em viveiro). Isabel Borges was funded by a PhD grant from Fundação para a Ciência e a Tecnologia (FCT) (POCI 2010).

## Sampling methods

### Study extent

Five Islands of the Azores (Portugal): São Miguel, Graciosa, Faial, Pico and So Jorge.

### Sampling description

The sampling programme in Faial, Graciosa, Pico and São Jorge consisted of travelling through each Island by car, for 3 to 4 days depending on the size of the Island. For São Miguel, we also included results taken in 2012 (Borges et al. 2011) in which fieldwork included a similar sampling effort. The samplings took place in representative habitats of the vegetation cover of the Islands that are visited by ladybeetles. The methods used to collect the samples were sweeping, beating and direct observations. Sampling from the herbaceous plants and canopy up to a height of ca. 3 m was standardised by using a standard sweep net (35-cm diameter, 140-cm handle) operated by António O. Soares, Isabel Borges and Hugo R. Calado. Independently of the method, the sampling effort was standardised in terms of the number of persons per unit of time (e.g. 1 person per 2 hours, 1 person per 30’, 1 person per 15'). Fieldwork occurred between 09:00 h and 16 :00 h on sunny and calm days. Ladybeetle adults were identified immediately and were released at the site and *Scymnus* spp were brought back to laboratory to identification.

## Geographic coverage

### Description

Azores Islands (Portugal): Faial, Graciosa, Pico, São Jorge and São Miguel

### Coordinates

36.906 and 39.589 Latitude; -24.961 and -31.311 Longitude.

## Taxonomic coverage

### Description

The sampling programme targeted labybeetles (Coleoptera: Coccinellidae)

### Taxa included

**Table:** 

Rank	Scientific Name	Common Name
family	Coccinellidae	Ladybeetles/ ladybirds/ ladybird beetles/ ladybugs

## Traits coverage

There are no trait data associated.

## Temporal coverage

### Notes

20 April 2012 to 6 July 2020

## Collection data

### Collection name

Ladybeetles of the Azores

### Collection identifier

ladybeetles

### Specimen preservation method

Ethanol 96%

### Curatorial unit

University of the Azores, Faculty of Sciences and Technology

## Usage licence

### Usage licence

Creative Commons Public Domain Waiver (CC-Zero)

## Data resources

### Data package title

Biodiversity data of ladybeetles (Coleoptera: Coccinellidae) of the Azores Archipelago (Portugal)

### Resource link

https://www.gbif.org/dataset/2292e622-129e-4c66-9ad6-fccaa377ff58

### Alternative identifiers

http://ipt.gbif.pt/ipt/resource?r=coccinellidae_azores&v=1.5

### Number of data sets

2

### Data set 1.

#### Data set name

Table of Sampling Events

#### Data format

Darwin Core Archive

#### Number of columns

28

#### Download URL

http://ipt.gbif.pt/ipt/resource?r=coccinellidae_azores&v=1.5

#### Data format version

version 1.5

#### Description

The following data table includes all the records for which a taxonomic identification of the species was possible. The dataset submitted to GBIF (Global Biodiversity Information Facility) is structured as a sample event dataset, with two tables: in the current event table, the data in this sampling event resource have been published as a Darwin Core Archive (DwCA), which is a standardised format for sharing biodiversity data as a set of one or more data tables. The core data file contains 98 records (eventID). This IPT (integrated publishing toolkit) archives the data and thus serves as the data repository. The data and resource metadata are available for download from Soares et al. (2021a).

**Data set 1.** 

Column label	Column description
id	Unique identification code for species abundance data. Equivalent here to eventID.
eventID	Identifier of the events, unique for the dataset.
samplingProtocol	The sampling protocol used to capture the species.
samplingEffort	The numeric amount of time spent in each sampling.
eventDate	Date or date range the record was collected.
year	Year of the event.
month	Month of the event.
day	Day of the event.
habitat	The habitat of the sample.
fieldNumber	An identifier given to the event in the field. Serves here as a link between field notes and the Event.
locationID	Identifier of the location.
islandGroup	Name of archipelago.
island	Name of the island.
country	Country of the sampling site.
countryCode	ISO code of the country of the sampling site.
stateProvince	Name of the region of the sampling site.
municipality	Municipality of the sampling site.
locality	Name of the locality.
verbatimLocality	The original textual description of the place.
maximumElevationInMetres	The upper limit of the range of elevation (altitude, usually above sea level), in metres.
locationRemarks	Details on the locality site.
verbatimCoordinates	The verbatim original spatial coordinates of the Location.
decimalLatitude	Approximate centre point decimal latitude of the field site in GPS coordinates.
decimalLongitude	Approximate centre point decimal longitude of the field site in GPS coordinates.
geodeticDatum	The ellipsoid, geodetic datum or spatial reference system (SRS) upon which the geographic coordinates given in decimalLatitude and decimalLongitude are based.
coordinateUncertaintyInMetres	Uncertainty of the coordinates of the centre of the sampling plot.
coordinatePrecision	Precision of the coordinates.
georeferenceSources	A list (concatenated and separated) of maps, gazetteers or other resources used to georeference the Location, described specifically enough to allow anyone in the future to use the same resources.

### Data set 2.

#### Data set name

Table of Species Occurrence

#### Data format

Darwin Core

#### Number of columns

29

#### Download URL

http://ipt.gbif.pt/ipt/resource?r=coccinellidae_azores&v=1.5

#### Data format version

version1.5

#### Description

The following data table includes all the records for which a taxonomic identification of the species was possible. The dataset submitted to GBIF (Global Biodiversity Information Facility) is structured as a sample event dataset, with two tables: in the current occurrences table, the data in this sampling event resource have been published as a Darwin Core Archive (DwCA), which is a standardised format for sharing biodiversity data as a set of one or more data tables. The core data file contains 218 records (occurrenceID). This IPT (integrated publishing toolkit) archives the data and thus serves as the data repository. The data and resource metadata are available for download from Soares et al. (2021a).

**Data set 2.** 

Column label	Column description
id	Unique identification code for species abundance data. Equivalent here to eventID.
type	Type of the record, as defined by the Public Core standard.
licence	Reference to the licence under which the record is published.
institutionID	The identity of the institution publishing the data.
collectionID	The identity of the collection publishing the data.
institutionCode	The code of the institution publishing the data.
collectionCode	The code of the collection where the specimens are conserved.
datasetName	Name of the dataset.
basisOfRecord	The nature of the data record.
occurrenceID	Identifier of the record, coded as a global unique identifier.
recordedBy	A list (concatenated and separated) of names of people, groups or organisations who performed the sampling in the field.
individualCount	A number or enumeration value for the quantity of organisms.
organismQuantityType	The type of quantification system used for the quantity of organisms.
lifeStage	The life stage of the organisms captured.
establishmentMeans	The process of establishment of the species in the location, using a controlled vocabulary: 'native', 'introduced', 'endemic', "uncertain".
eventID	Identifier of the events, unique for the dataset.
identifiedBy	A list (concatenated and separated) of names of people, groups or organisations who assigned the Taxon to the subject.
dateIdentified	The date on which the subject was determined as representing the Taxon.
identificationRemarks	Comments or notes about the Identification.
scientificName	Complete scientific name including author and year.
kingdom	Kingdom name.
phylum	Phylum name.
class	Class name.
order	Order name.
family	Family name.
genus	Genus name.
specificEpithet	Specific epithet.
taxonRank	Lowest taxonomic rank of the record.
scientificNameAuthorship	Name of the author of the lowest taxon rank included in the record.

## Additional information

A total of of 1,487 specimens of Coccinellidae belonging to 19 species were sampled (see Table 2). The listed species are from one single sub-familiy (Coccinellinae) and four tribes; Chilocorini (one species), Coccidulini (11 species), Coccinellini (six species) and Noviini (one species). The number of species collected from each island differed; São Miguel (12 species), Graciosa (four species), Faial (four species), Pico (seven species) and São Jorge (seven species).

*Propyleaquatuordecimpunctata* (Linnaeus, 1758), despite being previously listed to the Azores, but without island details by Soares et al. (2021b), is now recorded for the first time to Faial island. *Oenopiadoublieri* (Mulsant, 1846) was recently recorded as new for the Azores by Borges et al. (2018) (Terceira Island in Paúl da Praia da Vitória) and now is recorded to an additional island (Faial). Three additional species, *Rhyzobiuslophanthae* (Blaisdell, 1892), Scymnus (Pullus) suturalis Thunberg 1795 and *Stethoruspusillus* (Herbst, 1797), are new records to Pico, S. Miguel and Graciosa Islands, respectively.

Currently, the number of known species of ladybeetles in the Azores is 32 species (Soares et al. 2021b). The current list includes 30 confirmed species and two doubtful records (Table 3) and most of them considered exotic introduced species (n = 24) and only eight species are considered native. Two of the native species are endemic from the Macaronesian Region (*Nephusflavopictus* (Wollaston, 1854) and *Pharoscymnusdecemplagiatus* (Wollaston, 1857)) (see Table 3).

Doubtful records include *Eriopisconnexa* (Germar, 1824) and *Coccinellaseptempunctata* Linnaeus, 1758. We never collected these species in our extensive sampling programmes. With regard to *E.connexa*, it could result from misidentification given that this Neotropical species was never recorded outside its native region. In the case of *C.septempunctata*, although its previous presence in the Azores is well documented, its extinction may have occurred after the end of the cultural cycle of cereals, these being preferential habitats of the species (Soares et al. 2018).

The three Islands with highest economic activity are the ones with more species recorded (S. Miguel -22; Terceira - 16 and Faial - 13). The exception is Santa Maria that also has many species recorded (17), that can be explained by the proximity to S. Miguel and commercial exchanges between both Islands.

Interestingly, the same Islands are also the most diverse in the native fauna: S. Miguel - 7; Terceira - 6; Faial - 5; Santa Maria - 5. Only S. Jorge Island also has similar native species richness (five species) (Table 3).

Five alien species to the Palearctic Region were introduced in this region, as biological control agents of crop pests: *Delphastuscatalinae* (Horn, 1895), Nephus (Geminosipho) reunioni (Fürsch, 1974), *Noviuscardinalis* (Mulsant, 1850), *Rhyzobiusforestieri* (Mulsant, 1853) and *Rhyzobiuslophanthae* (Blaisdell, 1892) (Soares et al. 2021b).

The majority of the specimens was collected on herbaceous plants, including coastal prairies and ruderal road vegetation.

## Figures and Tables

**Table 1.** List of studied islands, habitats and localities with indication of elevation and geographical coordinates (datum WGS84).

**Island**	**Habitat**	**Locality**	**Elevation (m)**	**Latitude**	**Longitude**
Faial	Citrus orchard	Castelo Branco	57	38.5231	-28.68917
	Corn field	Cedros	166	38.62475	-28.68011
	Coastal mixed vegetation	Norte Pequeno	12	38.59263	-28.82711
	Coastal prairies	Pasteleiro	67	38.53005	-28.647701
	Coastal prairies	Praia do Almoxarife	5	38.5541	-28.61053
	Coastal prairies	Varadouro	8	38.56639	-28.77042
	Mixed forest of endemic and non-native host plants	Varadouro	175	38.57394	-28.77713
	Mixed forest of endemic and non-native host plants	Norte Pequeno	128	38.59433	-28.81541
	Ruderal road vegetation	Pasteleiro	93	38.53605	-28.64981
	Ruderal road vegetation	Varadouro	198	38.57952	-28.78283
	Urban poplar grove	Angústias	39	38.52806	-28.6367
	Vegetable garden	Feteira	37	38.52494	-28.68179
Graciosa	Abandoned vineyards	Beira Mar	10	39.02123	-28.00697
	Coastal prairies	Beira Mar	7	39.021	-28.00711
	Coastal prairies	Beira Mar	21	39.02373	-28.00686
	Coastal prairies	Sta. Cruz da Graciosa	25	39.09572	-28.03441
	Coastal Prairies, dominated by Canica sp.	Carapacho	17	39.01185	-27.97651
	Pasture: Medicago sativa L.	Jorge Gomes	58	39.0607	-28.06173
	Nerium oleander L. and Hibiscus rosa-sinensis L.	Alto do Sul	29	39.01192	-27.97911
	Ruderal road vegetation: herbaceous vegetation	Bom Jesus	9	39.08346	-28.05213
	Ruderal road vegetation: herbaceous vegetation	Bom Jesus	13	39.08189	-28.0542
	Ruderal road vegetation: herbaceous vegetation	Bom Jesus	19	39.08094	-28.05473
	Ruderal road vegetation: herbaceous vegetation	Jorge Gomes	69	39.06235	-28.06227
	Trees of Tamarix sp.	Bom Jesus	8	39.08376	-28.0524
	Vegetable garden	Porto da Barra	8	39.08469	-27.99925
Pico	Citrus orchard	Sete Cidades	119	38.52796	-28.50286
	Citrus orchard	Terra do Pão	55	38.42747	-28.40318
	Corn field	Monte	69	38.49832	-28.52976
	Corn field	São Vicente	113	38.54541	-28.36608
	Corn field	Sete Cidades	116	38.5286	-28.50279
	Coastal prairies	Madalena	3	38.52013	-28.53784
	Coastal prairies	Madalena	8	38.53957	-28.52029
	Evergreen forest	Toledos	15	38.54746	-28.50961
	Evergreen of endemic and exotic forest	Campo Raso	36	38.44743	-28.49908
	Pine trees	Sete Cidades	29	38.53353	-28.52339
	Pine trees	Sete Cidades	884	38.4976	-28.41566
	Ruderal road vegetation	Farrobo	114	38.54266	-28.42825
	Ruderal road vegetation: Arundo donax L.	Silveira	90	38.41783	-28.29147
	Ruderal road vegetation: Evergreen of endemic and exotic Forest	Cachorro	26	38.55574	-28.44033
	Ruderal road vegetation: Herbaceous plants	Cachorro	26	38.55574	-28.44033
	Ruderal road vegetation: Tamarix sp.	Madalena	3	38.52013	-28.53784
	Vegetable garden: cabbage	São Mateus	48	38.43294	-28.45794
	Vegetable garden: cabbage	São Vicente	113	38.54541	-28.36608
São Jorge	Citrus orchard	Fajã de S. Amaro	60	38.66226	-28.17184
	Citrus orchard	Fajã de S. Amaro	78	38.66261	-28.17155
	Coastal herbaceaous plants: Erica and Myrica	Portinho da Queimada	18	38.66651	-28.18714
	Coastal prairies	Queimada	14	38.67241	-28.19456
	Coastal prairies	Velas	27	38.6889	-28.2188
	Coastal prairies: Tamarix sp.	Velas	34	38.68693	-28.21873
	Vegetable garden: cabbage, bean and cucumber	Urzelina	60	38.64404	-28.1194
	Vegetable garden: cabbage, bean and cucumber	Velas	40	38.68181	-28.20469
	Wood: Acacia trees	Urzelina	59	38.64813	-28.12971
	Wood: Pinus trees	Urzelina	58	38.64383	-28.11937
São Miguel	Ruderal road vegetation: Arundo donax L.	Arrifres	130	37.75388	-25.70472
	Ruderal road vegetation: Arundo donax L.	Calhetas	18	37.82279	-25.61368
	Ruderal road vegetation: Arundo donax L.	São Roque	13	37.75152	-25.61896
	Ruderal road vegetation: Arundo donax L.	São Roque	13	37.75205	-25.62264
	Ruderal road vegetation: Arundo donax L.	São Roque	14	37.75205	-25.62264
	Coastal prairies	Fenais da Luz	30	37.83083	-25.635
	Coastal prairies	Pópulo	30	37.75023	-25.62106
	Coastal prairies	Rabo de Peixe	18	37.81583	-25.56694
	Coastal prairies	Rabo de Peixe	35	37.81378	-25.56706
	Coastal prairies	Relva	30	37.73737	-25.69819
	Coastal prairies	Relva	30	37.74711	-25.71359
	Coastal prairies	Santa Clara	30	37.7333	-25.686
	Coastal prairies	Santa Clara	30	37.73495	-25.69359
	Coastal prairies	São Roque	13	37.75152	-25.61896
	Corn field	Fenais da Luz	18	37.82666	-25.63194
	Corn field	Ribeira Seca	18	37.81659	-25.53795
	Corn field	São Sebastião	87	37.75424	-25.67236
	Pine trees	São Sebastião	76	37.75036	-25.67572
	Pine trees	São Sebastião	76	37.75036	-25.67572

**Table 2.** List of species with indication of number of individuals collected from each island. FAI - Faial; GRA - Graciosa; PIC - Pico; SJG - São Jorge; SMG - São Miguel. * refers to new species records for the island.

**Species**	**Tribe**	**FAI**	**GRA**	**PIC**	**SJG**	**SMG**	**Total**
*Adaliabipunctata* (Linnaeus, 1758)	Coccinellini	2	0	0	0	0	2
*Adaliadecempunctata* (Linnaeus, 1758)	Coccinellini	0	0	0	0	4	4
*Chilocorusbipustulatus* (Linnaeus, 1758)	Chilocorini	0	0	0	0	25	25
*Clitostethusarcuatus* (Rossi, 1794)	Scymnini	0	0	32	1	0	33
*Coccinellaundecimpunctata* Linnaeus, 1758	Coccinellini	0	0	0	0	7	7
*Myrrhaoctodecimguttata* (Linnaeus, 1758)	Coccinellini	0	0	3	0	0	3
Nephus (Geminosipho) reunioni (Fürsch 1974)	Scymnini	0	0	0	0	1	1
Nephus (Nephus) voeltzkowi Weise, 1910	Scymnini	8	2	0	1	147	158
*Noviuscardinalis* (Mulsant, 1850)	Noviini	5	0	29	6	0	40
*Oenopiadoublieri (Mulsant, 1846)	Coccinellini	6*	0	0	0	0	6
*Propyleaquatuordecimpunctata (Linnaeus, 1758)	Coccinellini	1*	0	0	0	0	1
*Rhyzobiuschrysomeloides* (Herbst, 1792)	Coccidulini	0	0	0	0	25	25
*Rhyzobiuslitura* (Fabricius, 1787)	Coccidulini	1	0	0	0	63	64
*Rhyzobiuslophanthae (Blaisdell, 1892)	Coccidulini	0	0	3*	3	0	6
Scymnus (Pullus) subvillosus (Goeze, 1777)	Scymnini	1	0	0	0	4	5
*Scymnus (Pullus) suturalis Thunberg 1795	Scymnini	0	0	0	0	6*	6
Scymnus (Scymnus) interruptus (Goeze, 1777)	Scymnini	20	21	22	25	322	410
Scymnus (Scymnus) nubilus Mulsant, 1850	Scymnini	66	149	218	35	180	648
*Stethoruspusillus (Herbst, 1797)	Stethorini	5	2*	26	6	4	43

**Table 3.** Current checklist (by alphabetic order) of the Azorean ladybeetles (Coleoptera: Coccinellidae). Doubtful records are marked with an asterisk (*). COL. – establishment means, in which INTRO is an exotic species introduced in the Azores, NAT is a native non-endemic species and MAC is an endemic species from Macaronesia. The names of the islands are as follows: AZ- recorded for Azores with no mention to the island; COR - Corvo; FLO - Flores; FAI - Faial; PIC - Pico; GRA - Graciosa; SJG - São Jorge; TER, Terceira; SMG - São Miguel; SMR - Santa Maria.

Scientific name	Col.	AZ	COR	FLO	FAI	PIC	GRA	SJG	TER	SMG	SMR
*Adaliabipunctata* (Linnaeus, 1758)	INTR				FAI					SMG	
*Adaliadecempunctata* (Linnaeus, 1758)	INTR		COR	FLO	FAI	PIC	GRA	SJG	TER	SMG	SMR
*Ceratomegillaundecimnotata* (Schneider, 1792)	INTR									SMG	
*Chilocorusbipustulatus* (Linnaeus, 1758)	INTR									SMG	SMR
*Clitostethusarcuatus* (Rossi, 1794)	INTR				FAI	PIC	GRA	SJG	TER	SMG	SMR
*Coccinellaseptempunctata* Linnaeus, 1758^*^	INTR								TER		SMR
*Coccinellaundecimpunctata* Linnaeus, 1758	INTR		COR	FLO	FAI	PIC	GRA	SJG	TER	SMG	SMR
*Delphastuscatalinae* (Horn, 1895)	INTR									SMG	
*Eriopisconnexa* (Germar, 1824)*	INTR									SMG	
*Hippodamiavariegata* (Goeze, 1777)	INTR	AZ									
*Myrrhaoctodecimguttata* (Linnaeus, 1758)	INTR									SMG	SMR
Nephus (Bipunctatus) bisignatus (Boheman, 1850)	INTR										SMR
Nephus (Geminosipho) reunioni (Fürsch, 1974)	INTR									SMG	
Nephus (Nephus) flavopictus (Wollaston, 1854)	MAC							SJG	TER	SMG	
Nephus (Nephus) voeltzkowi Weise, 1910	INTR		COR				GRA	SJG	TER	SMG	
*Noviuscardinalis* (Mulsant, 1850)	INTR		COR	FLO	FAI	PIC	GRA	SJG	TER	SMG	SMR
*Oenopiadoublieri* (Mulsant, 1846)	INTR				FAI				TER		
*Pharoscymnusdecemplagiatus* (Wollaston, 1857)	MAC	AZ									
*Propyleaquatuordecimpunctata* (Linnaeus, 1758)	INTR				FAI						
*Rhyzobiuschrysomeloides* (Herbst, 1792)	NAT									SMG	
*Rhyzobiusforestieri* (Mulsant, 1853)	INTR									SMG	
*Rhyzobiuslophanthae* (Blaisdell, 1892)	INTR			FLO		PIC	GRA	SJG	TER	SMG	SMR
*Rhyzobiuslitura* (Fabricius, 1787)	NAT				FAI		GRA		TER	SMG	SMR
*Scymniscushelgae* (Fürsch, 1965)	INTR			FLO					TER		SMR
Scymnus (Neopullus) haemorrhoidalis Herbst, 1797	INTR										SMR
Scymnus (Pullus) subvillosus (Goeze, 1777)	NAT				FAI			SJG	TER	SMG	SMR
Scymnus (Pullus) suturalis Thunberg, 1795	INTR				FAI				TER	SMG	SMR
Scymnus (Scymnus) interruptus (Goeze, 1777)	NAT		COR	FLO	FAI	PIC	GRA	SJG	TER	SMG	SMR
Scymnus (Scymnus) nubilus Mulsant, 1850	NAT		COR	FLO	FAI	PIC	GRA	SJG	TER	SMG	SMR
Scymnus (Scymnus) rubromaculatus (Goeze, 1777)	NAT	AZ									
Scymnus (Scymnus) schmidti Fürsch, 1958	INTR	AZ									
*Stethoruspusillus* (Herbst, 1797)	NAT			FLO	FAI	PIC	GRA	SJG	TER	SMG	SMR

## References

[B7489040] Alaniz Alberto J., Soares A. O., Vergara Pablo M., Azevedo Eduardo Brito, Grez Audrey A. (2020). The failed invasion of *Harmoniaaxyridis* in the Azores, Portugal: Climatic restriction or wrong population origin?. Insect Science.

[B7488732] Ameixa Olga Maria Correia Chitas, Soares António Onofre, Soares Amadeu M. V.M., Lillebø Ana I. (2018). Ecosystem services provided by the little things that run the world. Selected Studies in Biodiversity.

[B7489050] Bidinger K., Lötters S., Rödder D., Veith M. (2010). Species distribution models for the alien invasive Asian Harlequin ladybird (Harmoniaaxyridis). Journal of Applied Entomology.

[B7562014] Borges Isabel, Soares António Onofre, Magro Alexandra, Hemptinne Jean-Louis (2011). Prey availability in time and space is a driving force in life history evolution of predatory insects. Evolutionary Ecology.

[B7495150] Borges P. A. V., Gabriel Rosalina, Pimentel César, Brito Mariana, Serrano A. R. M., Crespo L. C., Assing Volker, Stüben Peter, Fattorini Simone, Soares A. O., Mendonça Enésima, Nogueira Elisabete (2018). Biota from the coastal wetlands of Praia da Vitória (Terceira, Azores, Portugal): Part 1 - Arthropods. Biodiversity Data Journal.

[B7489068] Cardoso Pedro, Erwin Terry L., Borges Paulo A. V., New Tim R. (2011). The seven impediments in invertebrate conservation and how to overcome them. Biological Conservation.

[B7488749] Cardoso Pedro, Barton Philip S., Birkhofer Klaus, Chichorro Filipe, Deacon Charl, Fartmann Thomas, Fukushima Caroline S., Gaigher René, Habel Jan C., Hallmann Caspar A., Hill Matthew J., Hochkirch Axel, Kwak Mackenzie L., Mammola Stefano, Ari Noriega Jorge, Orfinger Alexander B., Pedraza Fernando, Pryke James S., Roque Fabio O., Settele Josef, Simaika John P., Stork Nigel E., Suhling Frank, Vorster Carlien, Samways Michael J. (2020). Scientists' warning to humanity on insect extinctions. Biological Conservation.

[B7488796] Harmon Jason P., Stephens Erin, Losey John (2006). The decline of native coccinellids (Coleoptera: Coccinellidae) in the United States and Canada. Journal of Insect Conservation.

[B7488654] Harvey Jeffrey A., Heinen Robin, Armbrecht Inge, Basset Yves, Baxter-Gilbert James H., Bezemer T. Martijn, Böhm Monika, Bommarco Riccardo, Borges Paulo A. V., Cardoso Pedro, Clausnitzer Viola, Cornelisse Tara, Crone Elizabeth E., Dicke Marcel, Dijkstra Klaas-Douwe B., Dyer Lee, Ellers Jacintha, Fartmann Thomas, Forister Mathew L., Furlong Michael J., Garcia-Aguayo Andres, Gerlach Justin, Gols Rieta, Goulson Dave, Habel Jan-Christian, Haddad Nick M., Hallmann Caspar A., Henriques Sérgio, Herberstein Marie E., Hochkirch Axel, Hughes Alice C., Jepsen Sarina, Jones T. Hefin, Kaydan Bora M., Kleijn David, Klein Alexandra-Maria, Latty Tanya, Leather Simon R., Lewis Sara M., Lister Bradford C., Losey John E., Lowe Elizabeth C., Macadam Craig R., Montoya-Lerma James, Nagano Christopher D., Ogan Sophie, Orr Michael C., Painting Christina J., Pham Thai-Hong, Potts Simon G., Rauf Aunu, Roslin Tomas L., Samways Michael J., Sanchez-Bayo Francisco, Sar Sim A., Schultz Cheryl B., Soares António O., Thancharoen Anchana, Tscharntke Teja, Tylianakis Jason M., Umbers Kate D. L., Vet Louise E. M., Visser Marcel E., Vujic Ante, Wagner David L., WallisDeVries Michiel F., Westphal Catrin, White Thomas E., Wilkins Vicky L., Williams Paul H., Wyckhuys Kris A. G., Zhu Zeng-Rong, de Kroon Hans (2020). International scientists formulate a roadmap for insect conservation and recovery. Nature Ecology & Evolution.

[B7488788] Hodek Ivo, Honĕk A., van Emden H. F. (2012). Ecology and behaviour of the ladybird beetles (Coccinellidae).

[B7488830] Honĕk Alois, Martinkova Zdenka, Dixon Anthony F. G., Roy Helen E., Pekár Stano (2016). Long‐term changes in communities of native coccinellids: population fluctuations and the effect of competition from an invasive non‐native species. Insect Conservation and Diversity.

[B7488915] Honĕk Alois, Dixon Anthony FG, Soares Antonio O, Skuhrovec Jiri, Martinkova Zdenka (2017). Spatial and temporal changes in the abundance and compostion of ladybird (Coleoptera: Coccinellidae) communities. Current Opinion in Insect Science.

[B7490498] IPBES (2019). Summary for policymakers of the global assessment report on biodiversity and ecosystem services of the Intergovernmental Science-Policy Platform on Biodiversity and Ecosystem Services. S. Díaz, J. Settele, E. S. Brondízio, H. T. Ngo, M. Guèze, J. Agard, A. Arneth, P. Balvanera, K. A. Brauman, S. H. M. Butchart, K. M. A. Chan, L. A. Garibaldi, K. Ichii, J. Liu, S. M. Subramanian, G. F. Midgley, P. Miloslavich, Z. Molnár, D. Obura, A. Pfaff, S. Polasky, A. Purvis, J. Razzaque, B. Reyers, R. Roy Chowdhury, Y. J. Shin, I. J. Visseren-Hamakers, K. J. Willis, and C. N. Zayas (Eds)..

[B7495285] Magro Alexandra, Lecompte Emilie, Hemptinne Jean‐Louis, Soares Antonio O., Dutrillaux Anne‐Marie, Murienne Jérôme, Fürsch Helmut, Dutrillaux Bernard (2019). First case of parthenogenesis in ladybirds (Coleoptera: Coccinellidae) suggests new mechanisms for the evolution of asexual reproduction. Journal of Zoological Systematics and Evolutionary Research.

[B7489059] Poutsma J., Loomans A. J. M., Aukema B., Heijerman T. (2008). Predicting the potential geographical distribution of the harlequin ladybird, *Harmoniaaxyridis*, using the CLIMEX model. From Biological Control to Invasion: the Ladybird Harmoniaaxyridis as a Model Species.

[B7488805] Roy Helen E., Adriaens Tim, Isaac Nick J. B., Kenis Marc, Onkelinx Thierry, Martin Gilles San, Brown Peter M. J., Hautier Louis, Poland Remy, Roy David B., Comont Richard, Eschen René, Frost Robert, Zindel Renate, Van Vlaenderen Johan, Nedvěd Oldřich, Ravn Hans Peter, Grégoire Jean-Claude, de Biseau Jean-Christophe, Maes Dirk (2012). Invasive alien predator causes rapid declines of native European ladybirds. Diversity and Distributions.

[B7488840] Roy Helen E., Brown Peter M. J., Adriaens Tim, Berkvens Nick, Borges Isabel, Clusella-Trullas Susana, Comont Richard F., De Clercq Patrick, Eschen Rene, Estoup Arnaud, Evans Edward W., Facon Benoit, Gardiner Mary M., Gil Artur, Grez Audrey A., Guillemaud Thomas, Haelewaters Danny, Herz Annette, Honĕk Alois, Howe Andy G., Hui Cang, Hutchison William D., Kenis Marc, Koch Robert L., Kulfan Jan, Lawson Handley Lori, Lombaert Eric, Loomans Antoon, Losey John, Lukashuk Alexander O., Maes Dirk, Magro Alexandra, Murray Katie M., Martin Gilles San, Martinkova Zdenka, Minnaar Ingrid A., Nedvĕd Oldřich, Orlova-Bienkowskaja Marina J., Osawa Naoya, Rabitsch Wolfgang, Ravn Hans Peter, Rondoni Gabriele, Rorke Steph L., Ryndevich Sergey K., Saethre May-Guri, Sloggett John J., Soares Antonio Onofre, Stals Riaan, Tinsley Matthew C., Vandereycken Axel, van Wielink Paul, Viglášová Sandra, Zach Peter, Zakharov Ilya A., Zaviezo Tania, Zhao Zihua (2016). The harlequin ladybird, Harmoniaaxyridis: global perspectives on invasion history and ecology. Biological Invasions.

[B7488779] Seago Ainsley E., Giorgi Jose Adriano, Li Jiahui, Ślipiński Adam (2011). Phylogeny, classification and evolution of ladybird beetles (Coleoptera: Coccinellidae) based on simultaneous analysis of molecular and morphological data. Molecular Phylogenetics and Evolution.

[B7488936] Soares António O., Honěk Alois, Martinkova Zdenka, Brown Peter M. J., Borges Isabel (2018). Can native geographical range, dispersal ability and development rates predict the successful establishment of alien ladybird (Coleoptera: Coccinellidae) species in Europe?. Frontiers in Ecology and Evolution.

[B7488926] Soares António Onofre, Borges Isabel, Borges Paulo A. V., Labrie Geneviève, Lucas Eric (2008). Harmoniaaxyridis: What will stop the invader?. BioControl.

[B7488946] Soares António Onofre, Honěk Alois, Martinkova Zdenka, Skuhrovec Jiri, Cardoso Pedro, Borges Isabel (2017). Harmoniaaxyridis failed to establish in the Azores: the role of species richness, intraguild interactions and resource availability. BioControl.

[B7484312] Soares A. O., Borges I., Calado H., Borges P. A. V. (2021). Biodiversity data of ladybeetles (Coleoptera: Coccinellidae) of the Azores Archipelago (Portugal). v.1.5. http://ipt.gbif.pt/ipt/resource?r=coccinellidae_azores&v=1.5.

[B7488901] Soares António Onofre, Calado Hugo Renato, Franco José Carlos, Aguiar António Franquinho, Andrade Miguel M., Zina Vera, Ameixa Olga M. C. C., Borges Isabel, Magro Alexandra (2021). An annotated checklist of ladybeetle species (Coleoptera, Coccinellidae) of Portugal, including the Azores and Madeira Archipelagos. ZooKeys.

